# Impact of COVID-19 on maxillofacial surgery practice: a systematic review

**DOI:** 10.1016/j.bjorl.2021.09.002

**Published:** 2021-10-23

**Authors:** Luís Eduardo Charles Pagotto, Thiago de Santana Santos, Gabriel Pires Pastore

**Affiliations:** a Sírio Libanês Hospital, Instituto de Educação e Pesquisa (IEP), São Paulo, SP, Brazil; b Instituto de Educação Maxilofacial, Aracaju, SE, Brazil; c Sírio Libanês Hospital, Instituto de Educação e Pesquisa (IEP), Programa de Pós-Graduação em Ciências da Saúde, São Paulo, SP, Brazil

**Keywords:** COVID-19, SARS-CoV-2, Medical protection, Oral and maxillofacial surgery

## Abstract

**Objectives:**

The aim of this systematic review was to evaluate the possible impacts of COVID-19 on oral and maxillofacial surgery practice, as well as the protocols employed by oral and maxillofacial surgeons to minimize the risks of contamination.

**Methods:**

Searches were performed in different databases up to 15 March 2021. Articles related to oral surgery dynamics within the context of the COVID-19 pandemic were included. The articles were considered eligible if they met the following inclusion criteria: observational studies that provided recommendations regarding maxillofacial surgery measures during the COVID-19 pandemic and that analyzed the risk of contamination of patients/professionals with SARS-CoV-2.

**Results:**

Seven studies met the inclusion criteria and were selected for the present systematic review. Demands for personal protective equipment increased significantly, with studies reporting the scarcity of fundamental equipment such as FFP2/N95 masks. Significant changes in the infrastructure of outpatient, surgical and inpatient units and in the care protocols themselves were observed.

**Conclusion:**

The COVID-19 pandemic particularly affected the routine of oral and maxillofacial surgery residents. Several additional recommendations for surgical practice or for the surgical team were evaluated and discussed, such as performing primarily urgent and emergency surgeries, remote patient care and screening, postponing elective surgeries, and new teaching methods in oral and maxillofacial surgery residency programs. In addition, screening of patients and of the surgery team for COVID-19 is strongly recommended.

## Highlights

• COVID-19 directly affected the routine of oral and maxillofacial professionals.
• Demands for personal protective equipment increased significantly.
• A high prevalence of anxiety was observed among professionals during the COVID-19.

## Introduction

In December 2019, an outbreak of a new Coronavirus Disease (COVID-19) caused by Severe Acute Respiratory Syndrome Coronavirus 2 (SARS-CoV-2) was reported in Wuhan, China, which has become one of the greatest global health issues of this century.^1^ The rapid spread of SARS-CoV-2 has quickly become one of the greatest challenges for healthcare systems worldwide.^2^

The pathogen is predominantly transmitted through respiratory droplets.^3^, ^4^ In addition, SARS-CoV-2 was detected in saliva samples, a fact rendering saliva a potential route of transmission of COVID-19.^1^, ^2^ Health professionals are a risk group and are among those most affected by COVID-19, accounting for 29% of all diagnosed cases.^5^ Inevitably, health professionals come in close contact with infected patients, increasing the risk of professional/patient contamination.

Health services have been severely affected by the global pandemic and oral and maxillofacial surgery is no exception. Oral and maxillofacial surgeons must be aware of the new challenges posed by the risk of transmission of the virus between patients and the healthcare team.^6^ During the SARS-CoV-2 pandemic, professionals must organize the treatment of patients so that the risk of transmission of the infection is reduced as far as possible, while all relevant treatment options are available to provide adequate patient care.^7^ In addition, a programmatic system must exist for situations in which there may be a lack of availability of surgical personnel due to COVID-19 infection. Patient screening methods must be developed according to the degree of urgency of oral and maxillofacial surgery treatment.^7^

The aim of this systematic review was to evaluate the possible impacts of COVID-19 on oral and maxillofacial surgery practice, as well as the protocols employed by oral and maxillofacial surgeons to minimize the risks of contamination.

## Methods

This meta-analysis was conducted according to the Preferred Reporting Items for Systematic Reviews and Meta-Analyses (PRISMA) guidelines.^8^ The study was registered with PROSPERO under number CRD42021241303.

### Search strategy and study selection

Searches were performed in the PubMed/Medline, Web of Science, Science Direct, Scopus, Embase and Cochrane Collaboration Library databases (last update in March 2021) using the following search terms: (i) (“oral surgery” * OR “maxillofacial surgery” *) AND (“COVID-19” OR coronavirus OR “SARS-CoV-2” OR “2019-nCoV” OR “corona virus” OR “COVID”); (ii) ((“Oral surgery” * OR “maxillofacial surgery” *) AND (“COVID-19” OR coronavirus OR “SARS-CoV-2” OR “2019-nCoV” OR “corona virus” OR “COVID” AND ) AND Clinical practice).

In addition, the reference lists of eligible articles were hand searched. Duplicates were identified and removed. The search was performed without time or language restrictions. Manuscripts not originally published in English were translated for subsequent assessment.

The following eligibility criteria were applied according to the PICO framework for selection of the studies: (1) Patients: maxillofacial professionals and patients attending oral and maxillofacial surgery services during the COVID-19 pandemic; (2) Intervention: analysis of the risk of contamination with COVID-19 in oral and maxillofacial surgery settings and methods used to prevent this risk; (3) Control: group of COVID-19-negative patients/professionals; (4) Outcome: rate of cases diagnosed with COVID-19. The articles were considered eligible if they met the following inclusion criteria: observational studies that provided recommendations regarding maxillofacial surgery measures during the COVID-19 pandemic and that analyzed the risk of contamination of patients/professionals with SARS-CoV-2. Review articles, conference abstracts, editorials, or letters were excluded from the systematic review.

### Data extraction and quality assessment

The titles and abstracts of studies were retrieved using the search strategy and then selected independently by two authors. The complete content of the extracted studies was independently assessed for eligibility by the same two authors. Any situations that resulted in disagreement were resolved through a consensus meeting with a third reviewer. The following data were extracted: first author; time of publication; country of origin; study design; age, sex and number of patients; type of maxillofacial surgery performed; protocol used to prevent COVID-19; result of screening for COVID-19 (COVID-19/no COVID-19) before and after the surgical procedure; number of cases diagnosed with COVID-19 among patients and maxillofacial surgeons; complication (yes/no) of cases diagnosed with COVID-19, and type of complications.

The system for classifying evidence of the Oxford Centre for Evidence-Based Medicine (CEBM) was used to evaluate the level of evidence of each recommendation. The CEBM system categorizes the level of evidence from level 1 (highest level) to level 5 (lowest level).^9^ The Grading of Recommendations, Assessment, Development and Evaluations (GRADE) system was used to assess the quality of the selected studies.^10^

## Results

### Study selection

**Figure 1:**
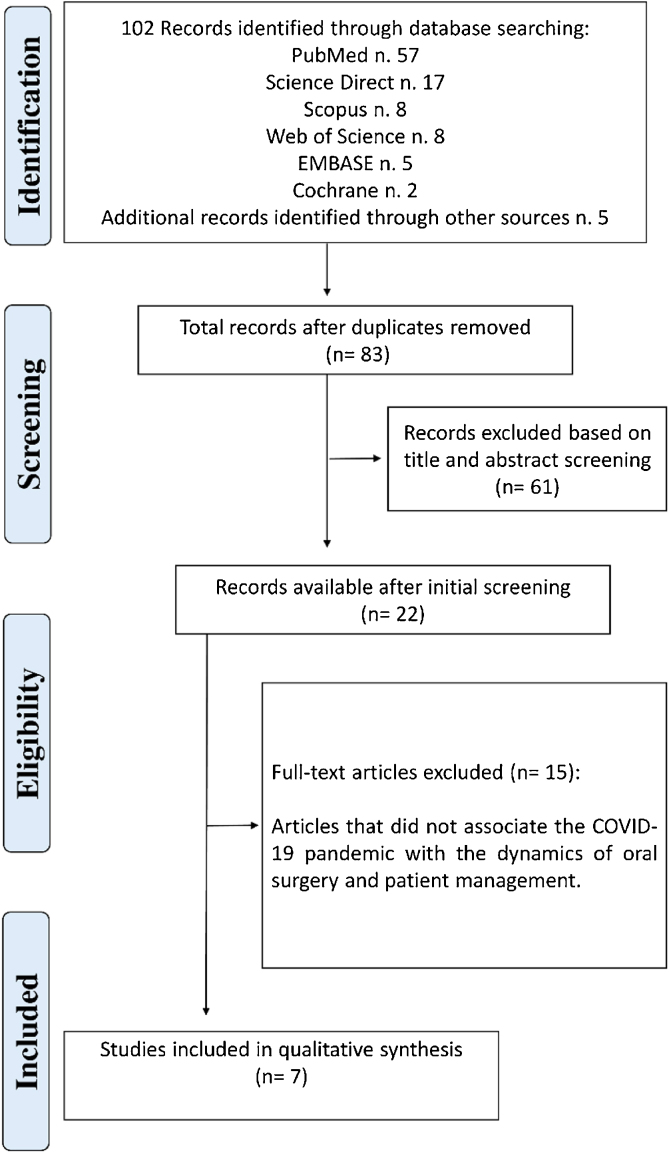
PRISMA flow diagram of screened studies.

The search strategy developed for the different databases and the hand search identified a total of 102 articles. Twenty-two articles were pre-selected after initial screening through analysis of the titles and abstracts. Seven studies met the inclusion criteria and were selected for the present systematic review (Fig. 1).^11^, ^12^, ^13^, ^14^, ^15^, ^16^, ^17^

These studies included data from 709 oral and maxillofacial surgeons, 562 patients, 174 oral and maxillofacial surgery and traumatology residents, and 95 residency program directors (Table 1). The quality of the selected studies was analyzed.

**Table 1:** Summary of findings from 7 studies included in the systematic review.

Study (year)	Type of study	Sample	Procedures performed	Proposed management of maxillofacial patients	COVID-19 results	Conclusions
Allevi et al. (2020)^11^	Cross-sectional survey	32 oral and maxillofacial surgeons	Most of maxillofacial surgery units have carried on the surgical management of facial trauma (74%) and head and neck oncology (90%).	Nasopharyngeal swabs were performed mostly in symptomatic patients (43%), followed by already hospitalized patients (18%) and candidates for surgery (9%). FFP2/N95 masks were provided in 61% of maxillofacial departments. Disposable gloves and surgical masks were provided in 91% and in 100% of maxillofacial wards, respectively, while disposable gowns were supplied only in 39% of maxillofacial units.	22% of maxillofacial units found infected surgeons (4% of maxillofacial surgeons), of different grades according to the intensity of contamination of the geographic areas.	All maxillofacial activity was greatly reduced during the period of the COVID-19 epidemic: tumor surgery and trauma surgery were largely guaranteed, while other pathologies accumulated delays.
Barca et al. (2020)^12^	Prospective analysis	33 Patients	The main pathologies were traumas and non-differentiable oncological diseases, in particular 20 were fractures and 13 tumours	1. Screening questionnaire.	All patients were negative.	The authors recommended:
						Repetition of triage;
				2. The nasopharyngeal swab (RT-PCR) was performed at the time of admission and after 24 h and the patients remained in dedicated areas until the results were available.		48 h of preoperative testing, before entering the ward, that includes two COVID-19 tests 24 h apart (if both tests are negative, then surgery can proceed with enhanced airborne precautions);
				3. Intraoperative protection: Healthcare staff used Personal protective equipment (PPE): N95 or FFP2 mask, eye protection, fluid-resistant gown, and surgical gloves.		Accommodation in a single hospital room;
						Speed of execution of the preoperative preparation
				4. Postoperative management: The patients remained hospitalised in individual rooms. They have undergone periodic control of the values of blood pressure, body temperature.		
Blackhall et al. (2020)^13^	Cross-sectional survey	529 patients	There were 255 trauma related cases, 221 infection and 48 cases of postoperative complications.	We found a relatively good use of PPE, appropriate for the clinical activity undertaken across the region. All clinicians who undertook surgical intervention within the oral cavity utilized the appropriate PPE including FFP3 masks. There was a varying use of PPE for examination only and extra-oral surgery, however there was no evidence to suggest inadequate protection.	The vast majority of the patients had an unknown COVID-19 status, however, were treated as if they were asymptomatic carriers of the infection.	Having a robust remote patient management system, and pre-planned pathway for managing dental emergencies would alleviate undue pressure on the service which needs to be established in partnership with various stakeholders involved in providing dental services.
Huntley et al. (2020)^14^	Cross-sectional survey	174 residency training	All the respondents indicated that their programs had made modifications to the scheduling of elective cases, with 97.7% stating their program had stopped performing elective cases altogether. Urgent or emergent cases were also affected, with 83.6% of respondents indicating that changes had been made to the scheduling of these cases.	Almost all residents (96.5%) reported modifications to their training program, and 14% had been reassigned to off-service clinical rotations (e.g., medicine, intensive care unit). The use of an N95 respirator mask plus standard PPE precautions during aerosol-generating procedures varied by procedure location, with 36.8% reporting limited access to these respirators. Widespread screening practices were in use, with 83.6% using laboratory-based viral testing.	NI	Sweeping alterations to oral and maxillofacial surgery clinical practice have occurred for those in Oral and maxillofacial surgery residency training programs during the COVID-19 pandemic.
Maffia et al. (2020)^15^	Cross-sectional survey	166 oral and maxillofacial surgeons	Traumatology was reported as the service that was most maintained, resulting in an OAI of 83.2%. Only 13.5% of the responding institutions had closed this subspecialty. Oral surgery, practiced in 90.4% of centers, decreased activity to 34.6%, with an overall reduction of 55.8% yielding and OAI of 38.3%.	Over half (57.1%) of the maxillofacial surgery centres that reported not receiving any COVID-19 management guidelines, did not receive personal protective equipment (PPE) from their administration either. Furthermore, 7% of the centres despite receiving such guidelines, received no PPE.	NI	It seems appropriate to request that every healthcare institution receives well-researched and documented protocols for dealing with future inevitable global pandemics.
van der Tas et al. (2020)^16^	Cross-sectional survey	511 oral and maxillofacial surgeons	More than 80% of all surgeons in the different regions stopped performing elective surgery.	The best protection offered to the surgeons is in Australia, where surgeons can work with N95/FFP2 masks (60.0%) or PAPR systems (40.0%), followed by North America reporting availability of N95/FFP2 masks (47.7%).	NI	The impact of COVID-19 among CMF surgeons is global and adversely affects both clinical practice and personal lives of craniomaxillofacial surgeons.
Brar et al. (2021)^17^	Cross-sectional survey	95 residency program directors	In the midst of the COVID-19 pandemic, all participating programs continued to provide limited patient services. These included emergency dental services (93.9%); emergent and urgent surgical procedures, such as repair of facial fractures or oncologic resection and reconstruction (93.9%); postoperative follow-up visits (75.8%); inpatient dental consultations (72.7%); and new outpatient consultations (36.4%).	In the operating room setting, the level of PPE recommended for aerosol-generating procedures involving the aerodigestive tract for use by patients of unknown COVID-19 status comprised primarily an N95 respirator with full-face shield, disposable gown, and gloves (61.5%).	NI	OMFS training programs should give more consideration to providing residents with adequate stress reduction resources to maintain their well-being and training and to minimize exposure risk during an evolving global epidemic.

NI, not informet.

### Operative impact and change in working patterns

All included studies reported a decrease in the number of surgical procedures through the cancellation/postponement of elective surgeries, with priority being given to urgency/emergency cases. In the study of Barca et al.^12^ the main surgeries performed during the emergency period caused by COVID-19 were those related to traumas and different oncological diseases. At the time of the pandemic, Blackhall et al.^13^ reported 255 cases related to trauma, 221 infections, and 48 cases of postoperative complications during the study period. Maffia et al.^15^ found traumatology to be the least affected area, which was maintained in 83.2% of all services of the interviewed professionals.

An important finding was the adaptation of consultations and patient screening. Barca et al.^12^ used a questionnaire for patient screening by telephone and on admission to the clinic, which consisted of the following topics: (1) Presence of fever in the last 14 days; (2) Possible respiratory problems; (3) Travel to risk areas with high COVID-19 spread; (4) Contact with COVID-19-positive patients, and (5) Participation in agglomerations. All patients underwent an RT-PCR test before the surgical procedure and were hospitalized in individual rooms.

Blackhall et al.^13^ attended 134 patients remotely by phone or video for initial screening on the risk of COVID-19 and the need for face-to-face health care. Huntley et al.^14^ reported that almost all programs (95.7%) in which residents participated had screened patients for symptoms of COVID-19. This was done more frequently by hospital screening (65.2%) or by the reception staff (62.2%). Among the respondents, 83.6% indicated that their program was using laboratory tests for patients with COVID-19.

Barca et al.^12^ reported that the healthcare team implemented preventive measures during the surgical procedure, as recommended by the provisional guidelines of the World Health Organization (WHO). The healthcare team used personal protective equipment: N95 or FFP2 masks, eye protection, fluid-resistant gowns, and surgical gloves. Blood pressure, body temperature, heart rate, and oxygen saturation were monitored periodically. No cases of COVID-19 were detected in the study. In an international multicenter study, Maffia et al.^15^ found that more than half (57.1%) of the maxillofacial surgery centers that reported not to have received any instructions on COVID-19 management did also not receive adequate personal protective equipment.

In the study of Brar et al.,^17^ 16 of the 33 programs (48.5%) did not report the lack of personal protective equipment, while 51.5% did. The most common missing devices were N95 respirators (94.1%) and surgical masks with face shield (35.3%). In a global study conducted by van der Tas et al.,^16^ the perception of the availability of personal protective equipment for health professional and surgeons differed significantly between the countries analyzed. The best protection offered to maxillofacial surgeons was observed in Australia, where surgeons could work with N95/FFP2 masks in 60.0% of services, followed by North America (47.7%).

### Educational/academic

In the study of Huntley et al.,^14^ all respondents indicated that their programs made changes to the scheduling of elective cases, with 97.7% of the interviewed residents reporting that their program had stopped elective procedures. Urgency or emergency cases were also affected, with 83.6% of respondents indicating changes in the scheduling of these cases. Regarding personal protective equipment, 95.3% of the respondents stated that their programs made changes in the use of this equipment. Most residents reported good (32.7%) or excellent (32.1%) access to masks with eye protection; however, 36.8% indicated limited access to N95 masks.

The study of Brar et al.^17^ found that all residency programs continued to provide limited services to patients during the COVID-19 pandemic. These included emergency dental treatment (93.9%); emergency and urgent surgical procedures such as the correction of facial fractures or oncological resection and reconstruction (93.9%); postoperative follow-up visits (75.8%); dental consultations with hospitalization (72.7%), and new outpatient appointments (36.4%).

Residents were strongly concerned about the possible practical effects of the pandemic on surgical teaching and training. Huntley et al.^14^ reported that residents were more concerned about their experience in orthognathic surgery (55.1%), ambulatory anesthesia and deep sedation (43.6%) and reconstructive and esthetic surgery (42.3%), as well as general experience in anesthesia (32.1%). Most residents (88.8%) indicated that their surgical experience was affected, with a mean reduction in surgical experience of 67%. The majority of respondents (94.2%) reported that their program used virtual didactics (online) and that the frequency of didactics had generally increased.

The COVID-19 pandemic has physically, mentally, and emotionally challenged residents, teachers, and staff. The study of Brar et al.^17^ indicates that different techniques for wellbeing were particularly important to maintain the health and safety of residents during this period, including meditation seminars, yoga routines and physical exercises, free or reduced fare transit passes, food delivery services, counseling services, and free hotel accommodation.

## Discussion

The results of this systematic review indicate that COVID-19 directly affected the routine of oral and maxillofacial professionals and referral centers around the world. Greater control of hospital procedures aimed at preventing the transmission of infection from patients to the healthcare team and vice-versa was observed in all studies.^12^, ^13^, ^14^, ^15^, ^17^ However, different studies reported the lack of personal protective equipment.^17^ We highlight studies that demonstrated that SARS-CoV-2 can penetrate traditional surgical masks and clothing, thus requiring the use of N95 masks and level 3 protective suits.^18^, ^19^ However, the scarcity of N95 masks is a global reality.^19^, ^20^

A high prevalence of anxiety was observed among professionals during the COVID-19 pandemic, especially among residents.^14^ Aziz et al.^21^ found similar results for general surgery residents in the United States; in addition, 33.1% of the respondents were diagnosed with burnout syndrome during the COVID-19 pandemic. Support mechanisms for maintaining mental health would be important for residents and professionals of postgraduate programs. Psychological monitoring and the encouragement of physical activities are useful for this purpose.

Furthermore, the use of remote teaching tools is fundamental during the period of social distancing to avoid educational neglect; in parallel, it is important to support concepts of technology-enhanced learning such as the use of active methodologies. According to Huntley et al.^14^ and Brar et al.,^17^ remote teaching was well accepted by residents and directors of oral and maxillofacial surgery referral centers, corroborating the findings of other studies in different areas.^21^, ^22^

Asymptomatic patients and those in the incubation period can be carriers of SARS-CoV-2 and can transmit the infection.^23^ Therefore, RT-PCR should be used as a screening method before the surgical procedure even in asymptomatic patients in order to monitor patient-surgical team and surgical team-patient contamination. Regular testing of professionals of the surgical team can also be used to avoid contamination.^24^

The present systematic review found that the surgical routine of urgency and emergency cases was the least affected, with the procedures being maintained. However, elective cases must be followed up periodically to assess the condition of the patients. This follow-up can be done by video calls with oral and maxillofacial professionals. Video calls can also be used as an initial screening method for possible symptoms associated with COVID-19 in patients who require a face-to-face consultation or surgery in the oral and maxillofacial surgery setting.^12^

**Figure 2:**
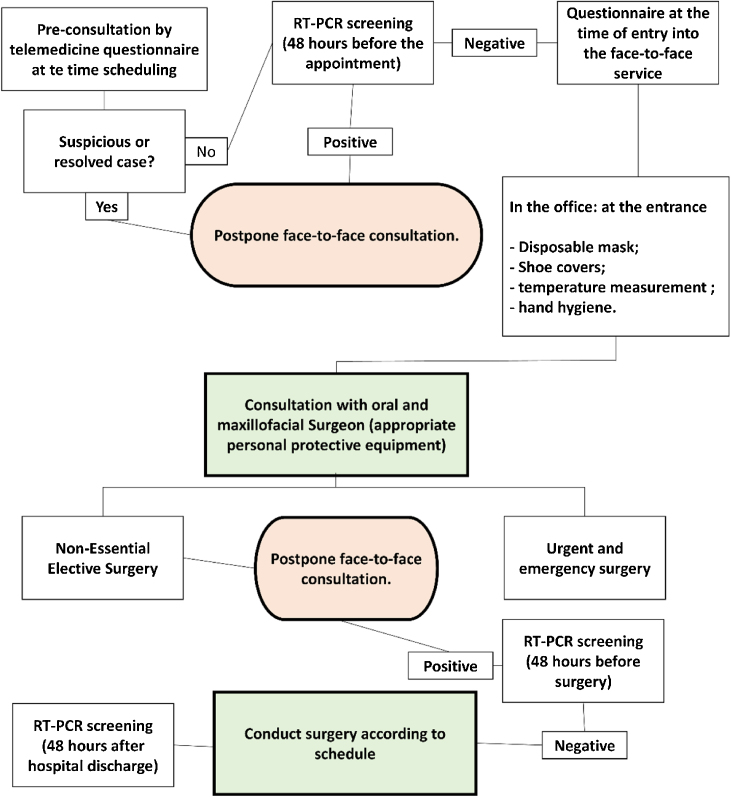
Flowchart proposed for oral and maxillofacial surgery due to COVID-19.

Symptomatic patients with COVID-19 should only be submitted to oral and maxillofacial surgery procedures in the case of an urgent or emergency situation. Symptomatic patients are the main source of viral transmission and must therefore be treated in an environment where an adequate infrastructure and personal protective equipment are available.^7^ We developed a flowchart for the management of patients in oral and maxillofacial surgery settings based on the recommendations of the different studies analyzed in this systematic review (Fig. 2).

The infrastructure of spaces of oral and maxillofacial surgery centers that receive inpatients and outpatients must be adjusted in such a way to avoid cross-contamination between outpatient and inpatient units. All patients who attend clinical departments must have their body temperature checked. However, patients infected with COVID-19 are not necessarily febrile. Initial screening is therefore also important to assess recent travel histories and contacts with infected patients. Before admission to the wards or before oral and maxillofacial surgery, the patients must be tested for the virus.^7^, ^25^

Healthcare workers are at the forefront of the fight against COVID-19 and are therefore considered a priority group in vaccination campaigns.^20^, ^26^ However, the vaccination rate varies widely between countries and the risk of emergence of new variants resistant to the different vaccines developed makes it necessary that protocols designed to reduce the risk of contamination with COVID-19 be maintained for an indefinite period of time.

## Conclusion

COVID-19 represents a challenge for oral and maxillofacial surgeons. Significant changes in the infrastructure of different outpatient, surgical and inpatient units and in the care protocols themselves were and will continue to be necessary in order to avoid unnecessary risks of contamination of patients and of the surgical team. Demands for personal protective equipment increased significantly, with reports of scarcity of fundamental equipment such as FFP2/N95 masks. The teaching methodology of oral and maxillofacial surgery residency programs must be adapted to the social distancing caused by the pandemic. In addition, psychological support should be offered to professionals and residents.

## Conflicts of interest

The authors declare no conflicts of interest.
